# ﻿On two new *Phyllidia* species (Gastropoda, Nudibranchia, Doridina) and some histology from the Coral Triangle

**DOI:** 10.3897/zookeys.1245.153046

**Published:** 2025-07-14

**Authors:** Heike Wägele, Lina Marie Raubold, Adelfia Papu, Nani Undap, Nathalie Yonow

**Affiliations:** 1 Leibniz Institute for the Analysis of Biodiversity Change, Museum Koenig Bonn, Adenauerallee 160, 53113 Bonn, Germany Museum Koenig Bonn Bonn Germany; 2 Richard Gilder Graduate School, Division of Invertebrate Zoology, American Museum of Natural History, 200 Central Park West, New York, NY 10024, USA American Museum of Natural History New York United States of America; 3 Faculty of Mathematics and Natural Sciences, Universitas Sam Ratulangi, Manado 95115, Indonesia Universitas Sam Ratulangi Manado Indonesia; 4 Research Center for Conservation of Marine and Inland Water Resources, National Research and Innovation Agency, Bogor 16911, Jawa Barat, Indonesia National Research and Innovation Agency Bogor Indonesia; 5 Biological Sciences, College of Science, Swansea University, Singleton Park, Swansea SA2 8PP, Wales, UK Swansea University Wales United Kingdom

**Keywords:** Biogeography, Indonesia, morphology, North Sulawesi, *Phyllidiaocellata*, Phyllidiidae, taxonomy

## Abstract

Two new species of *Phyllidia* from North Sulawesi, Indonesia, *Phyllidiafontjei***sp. nov.** and *Phyllidiaovata***sp. nov.**, are described based on morphology and molecular barcoding of CO1 and/or 16S. Both species are rare and distinctive and can be easily recognised by their colouration. Additionally, histological sections were made of the holotype of *Phyllidiafontjei***sp. nov.** and a similarly sized *Phyllidiaocellata* and these morphologies are compared with the only other detailed histological examination of the Mediterranean *Phyllidiaflava*.

## ﻿Introduction

The genus *Phyllidia* Cuvier, 1797, of the radula-less dorid family Phyllidiidae, currently comprises 29 acknowledged species (WoRMS 2025). Compared to the other phyllidiid genera, *Phyllidiopsis* (30 species), *Phyllidiella* (approximately 15 species), *Reticulidia* (4 species), and *Ceratophyllidia* (2 species), *Phyllidia* is the second species-rich genus (WoRMS 2025). All five genera show distinct morphological characters (Brunckhorst 1993; Yonow 2011), although anatomical differences among species within the genera are minimal. Identifications are often based on colour patterns, which can vary to a great extent in some species and are also not commonly investigated. Recent molecular studies on the family revealed several new species that are clearly distinct from the described species (Stoffels et al. 2016; Papu et al. 2022). Additionally, identification books (e.g., Gosliner et al. 2018) and networks (e.g., Sea Slug Forum 2010; NudiPixel 2025; iNaturalist 2025) have published photographs of animals that cannot be assigned to any described phyllidiid species. Usually, records of these new species are rare and therefore they are often not formally described. Here we describe two of these *Phyllidia* species as new to science, for which we collected new material that was adequately preserved for description and further analyses. *Phyllidiafontjei* sp. nov. is a small species, only similar to *Phyllidiamonacha* Yonow, 1986 (endemic to the Red Sea) and *Phyllidiakoehleri* Perrone, 2000 (endemic to the Maldives). The second new species, *Phyllidiaovata* sp. nov., is unique in its colouration and can also be identified by its external appearance. Due to its unique colouration, individuals that were previously assigned to *Phyllidiapicta* Pruvot-Fol, 1957 and *Phyllidiacoelestis* Bergh, 1905 on the Internet can now be correctly assigned to this new species.

*Phyllidiafontjei* sp. nov. was serially sectioned and the anatomical and histological results are compared with new histological investigations on *P.ocellata* Cuvier, 1804, which has previously been resolved as a sister taxon of our new species (Papu et al. 2022). Both are described herein and compared to *Phyllidiaflava* Aradas, 1847, the only other species for which comprehensive histological results have been published (Wägele 1985).

## ﻿Materials and methods

Material investigated in this study was collected by scuba diving on Bunaken Island (North Sulawesi, Indonesia) in 2015 (*Phyllidiaovata* sp. nov.) and 2017 (*Phyllidiafontjei* sp. nov.) and on Sangihe Island (North Sulawesi, Indonesia) in 2016 (*Phyllidiaocellata*). The three specimens were examined, measured, and photographed alive in bowls. For molecular barcoding (CO1 and 16S), a small piece of the posterior third of the body was cut off and preserved in 96% EtOH, and the remainder of the animals was preserved in 4% formaldehyde/seawater for further anatomical examination. The single animal of *Phyllidiafontjei* sp. nov. was completely preserved in 96% EtOH. Results on sequences of these three specimens are published in Undap et al. (2019: *P.ocellata*) and Papu et al. (2022: *Phyllidia* sp. a, *P.* sp. 9) and are available in GenBank: *Phyllidiafontjei* sp. nov. as *Phyllidia* sp. a with internal number Phsp17-Bu1 (only CO1: MZ964307), *Phyllidiaocellata* with internal number Phoc16Sa-4 (16S: MN173894, CO1: MN234124), and *Phyllidiaovata* sp. nov. with internal number Phsp15Bu-1 as *Phyllidia* sp. 9 (16S: MZ955502, CO1: MZ964198).

For anatomical analyses, the specimens of *P.fontjei* sp. nov. and *P.ocellata* were embedded in hydroxyethyl methacrylate (Heraeus Kulzer GmbH) for serial sectioning. Sections (2.5 µm thick) were stained with toluidine blue, subsequently photographed under a ZEISS Microscope (Imager.Z2m), and analysed with ZEN software (ZEISS). *Phyllidiaovata* sp. nov. was only investigated externally. The histological slides of *Phyllidiafontjei* sp. nov. (LIBHIS0004) and *Phyllidiaocellata* (LIBHIS00005) are stored in the histological data base of the Leibniz-Institute, Museum Koenig Bonn, Germany. The holotype of *P.ovata* is deposited in Bogor, Indonesia in the Museum Zoologicum Bogoriense (MZB.Gst.25761).

Species distribution maps were created in QGIS v. 3.34. Background map data (global cultural boundaries, 1:10 m) were downloaded from https://www.naturalearthdata.com/ and projected in ESPG:4326. For occurrences for which locality data but no coordinates were available, georeference data were inferred. For localities with multiple occurrences, all with unclear georeference data, occurrences were bundled. This refers to the Similan Islands (*n* = 2) and Surin Island (*n* = 3) for *P.fontjei*, as well as Bunaken Island (*n* = 2) and Wakatobi (*n* = 2) for *P.ovata*. Georeference data (latitude, longitude) for occurrences with published georeference data are listed under Distribution of each species.

## ﻿Results

### ﻿Taxonomy

#### ﻿ Phyllidia fontjei sp. nov.

Taxon classificationAnimaliaNudibranchiaPhyllidiidae

0E8F6F23-8C24-5C27-B1E0-BAB94EC7F4ED

https://zoobank.org/BDC62053-0951-4503-B255-77F46F451349

Fig. 1A–C

Phyllidia sp.: Eisenbarth et al. 2018: fig. 20a, b; same specimen (holotype) depicted and described as Phyllidia sp. a in Papu et al. 2022: fig. 6a (Bunaken Is., Indonesia).
Phyllidiopsis monacha Yonow: Gosliner et al. 2008: 300, photograph top right (Kepulauan Seribu, Indonesia).
non Phyllidiopsismonacha Yonow: Gosliner et al. 2008: 300, photograph top left (Eilat, Israel).

##### Type material.

***Holotype*** (serially sectioned): • Phsp17-Bu1 [LIBHIS00004], Panorama, Bunaken Island, North Sulawesi, Indonesia [1°36'50"N, 124°46'3.4"E], collected in September 2017 on a sponge at 25.3 m depth, 16 mm in length alive. The only available CO1 sequence (MZ964307) is published in Papu et al. (2022 as *Phyllidia* sp. a).

##### Diagnosis.

**Figure 1.**
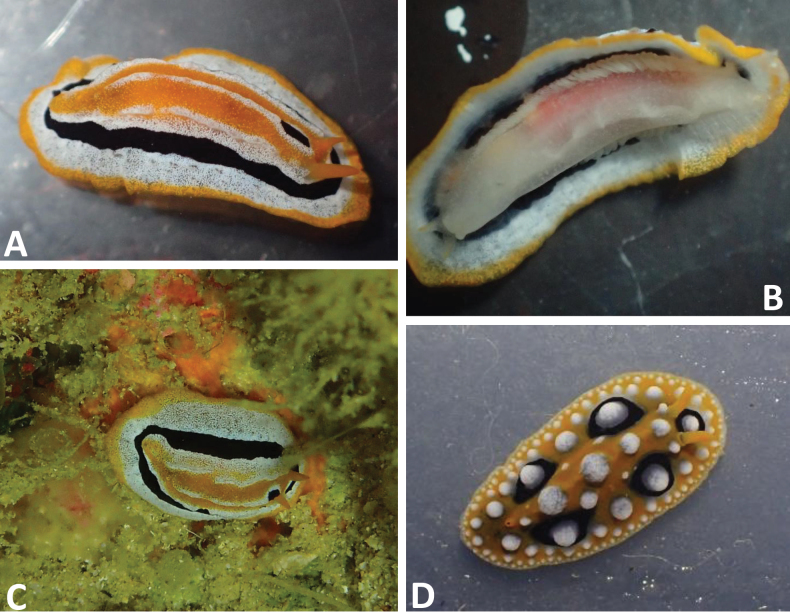
**A–C.***Phyllidiafontjei* sp. nov. holotype (Phsp17-Bu1, Panorama, Bunaken Island), living specimen 16 mm. **A.** Dorsal view; **B.** Ventrolateral view; **C.** In situ on a dark-orange sponge on the roof in a small cave at 25 m depth. Note that the colour of the sponge is similar to the coloration of the digestive tract showing through the ventral body in Fig. 1B; **D.***Phyllidiaocellata* (Phoc 16Sa-4, Sangihe Island), living specimen 16 mm.

The single specimen is white with a narrow, granulated, orange mantle margin followed by a wide white band, a narrower black line, and then a white ring that also forms an elevated ridge on its inner margin. The central part is orange and shows a prominent and elevated white central ridge (Fig. 1A). The holotype also has a black elongate mark in this central white ridge, which is missing in other individuals that were identified from images (see Distribution below). The species is not tuberculate but bears three ridges. Minute granules are present all over the white part of the dorsum. The yellow/orange rhinophores emerge from the inner white ring and bear 12 lamellae on each rhinophoral clavus. Rhinotubercles are absent. The dorsal anus lies near the posterior margin of the central orange part. Ventrally, the foot of *Phyllidiafontjei* sp. nov. has a white appearance and the oral tentacles are yellow-tipped. The hyponotum is rather transparent, and the dorsal orange and black lines show through (Fig. 1B), as does a reddish gut content that perhaps comes from the dark orange sponge on which the animal was probably feeding (Fig. 1C in situ).

##### Distribution.

Western Pacific Ocean: **Indonesia**: Bunaken Is., North Sulawesi (Eisenbarth et al. 2018; provided georeference: 1.61389, 124.76761); Timor Leste (https://nudipixel.net/photo/00032422); Lembeh (https://nudipixel.net/photo/00009806); Pulau Seribu (https://nudipixel.net/photo/00005577); Kepulauan Seribu (Gosliner et al. 2008: 300, photograph top right as *P.monacha*). **Malaysia**: (https://www.inaturalist.org/observations/177306163; provided georeference: 4.24570, 118.63158).

Indian Ocean: **Andaman Sea**: Similan Islands, Thailand (https://nudipixel.net/photo/00011938, https://www.facebook.com/photo/?fbid=657059971085019&set=a.219891531468534 [with tubercles]), Surin Islands (https://www.facebook.com/photo/?fbid=591593289670092&set=a.219891531468534 [tiny individual], https://www.facebook.com/photo/?fbid=539864628176292&set=a.219891531468534, https://www.facebook.com/photo/?fbid=539804631515625&set=a.219891531468534), Krabi (https://www.facebook.com/photo/?fbid=3501673139957007&set=a.219891531468534); Andaman and Nicobar (https://nudipixel.net/photo/00039161).

##### Remarks.

*Phyllidiafontjei* is most similar to *Phyllidiamonacha* Yonow, 1986 and *Phyllidiakoehleri* Perrone, 2000. *Phyllidiamonacha* is also a small yellow, black, and white species with ridges formed by minute tubercles on the dorsum. *Phyllidiafontjei* sp. nov. is illustrated by Gosliner et al. (2008: 300, top right) as *Phyllidiopsismonacha*. We assume that this individual was erroneously assigned to the genus *Phyllidiopsis* due to previous (also erroneous) synonymisation with *Phyllidiopsisdautzenbergi* (Vayssière, 1912) by Brunckhorst (1993); both species are discussed and illustrated by Yonow (2021). *Phyllidiamonacha* has distinct tubercles, which seem to be present (but less pronounced) in images of larger individuals of *P.fontjei. Phyllidiamonacha* has a central black irregular ring, similar to *P.fontjei* sp. nov.; however, in *P.monacha* there are black radiating lines extending from this black area towards the mantle margin and separately enclosing the rhinophores, which are each located on an individual white or yellowish patch. In *P.fontjei* sp. nov. there is only one simple black ring and the rhinophores lie in the yellow band within this black band.

*Phyllidiafontjei* sp. nov. is also similar to *Phyllidiakoehleri*, a completely yellow species with a bold black ring encircling the central dorsum that can break up into rays extending to the margin (e.g., Yonow 2012: 61, pl. 65), a median black line dorsally, and minute tubercles forming ridges centrally on the dorsum; however, *P.koehleri* lacks the white pigmentation present in both *P.fontjei* sp. nov. and *P.monacha*. Including a recently published CO1 sequence of this species (Cunha et al. 2023) in our *Phyllidia* dataset from Papu et al. (2022) renders this species a sister group to *P.fontjei*, with both being sister group to *P.ocellata*.

These three species are all small compared to many other species that may reach 60 mm and more (Gosliner et al. 2018), and all have restricted distributions: the maximum preserved lengths of *P.koehleri* and *P.monacha* are 17 mm and 14 mm, respectively; *P.fontjei* measured 16 mm alive. The first two species are endemic to the Maldives and the Red Sea, respectively, while *P.fontjei* is restricted to Indonesia, Malaysia, and the Andaman Sea (see Distribution records listed above). *Phyllidiafontjei* is distinctive and can be accurately identified from photographs.

##### Etymology.

We name this species after our dear colleague Prof. Dr. Fontje Kaligis from Sam Ratulangi University, Manado, who initiated our Indonesian co-operation on describing marine Heterobranchia diversity around North Sulawesi. He passed away in September 2017, too early to see all the publications resulting from the joint collecting efforts. He enabled the extensive biodiversity studies in North Sulawesi and thus greatly extended our understanding of sea slugs in this area.

#### ﻿ Phyllidia ovata sp. nov.

Taxon classificationAnimaliaNudibranchiaPhyllidiidae

F1CC7C9F-5DDD-5C03-9CD8-F98D777272E2

https://zoobank.org/D427254F-AECC-42DA-B602-5CBAB9D532F1

Fig. 2A, B

Phyllidia sp. 9: Gosliner et al. 2015: 282 (Philippines); Papu et al. 2022: fig. 5.4a (Bunaken Is., Indonesia, same specimen).
Phyllidia sp.: Stoffels et al. 2016: figs 4, 7G (Ternate, Indonesia).
Phyllidia sp. 10: Gosliner et al. 2018: 217 (same individual in Gosliner et al. 2015 as Phyllidia sp. 9).
Fryeria sp.: Kato 2002 (http://www.seaslugforum.net/find/6414), size 50 mm, 30 m depth (Japan).

##### Type material.

***Holotype***: • Phsp15Bu-1 (MZB.Gst.25761), Cela Cela, Bunaken Island, North Sulawesi, Indonesia [1°36'42.4"N, 124°46'4.7"E], collected 13 August 2015 at a depth of 16 m, 35 mm in length alive. Available CO1 (MZ964198) and 16S (MZ955502) sequences are published in Papu et al. (2022 as *Phyllidia* sp. 9).

##### Diagnosis.

**Figure 2.**
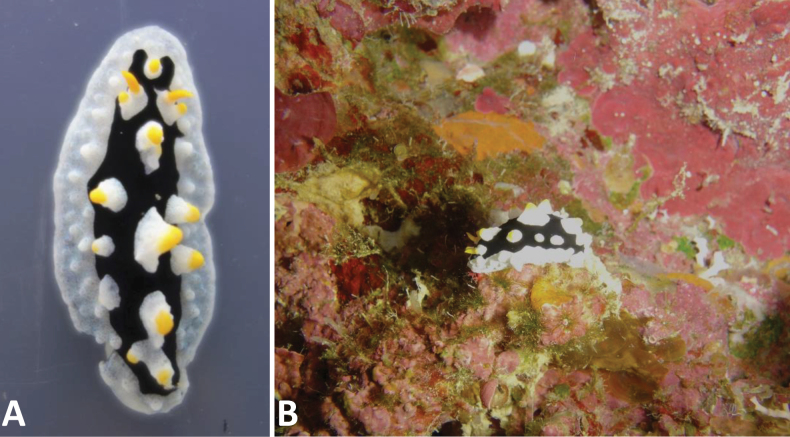
*Phyllidiaovata* sp. nov. holotype (Phsp15Bu-1, Cela Cela, Bunaken Island), living specimen 35 mm. **A.** Dorsal view; **B.** In situ on a reef wall overgrown with coralline algae, filamentous green algae, sponges, small hydroids, and serpulids.

The mantle is elongate oval, white (rarely with a tinge of blue) with a clean-margined oval black area covering the central notum. Anteriorly, this black pigment forms a rounded U-shape around the first midline tubercle placed in front of the rhinophores (Fig. 2A, B). Large white conical tubercles have yellow apices in the middle three rows and are only present in the black area. The broad white mantle skirt has smaller white tubercles decreasing in size towards the margin. Yellow rhinophores arise from translucent white rhinophoral sheaths that are distinctly raised; a rhinophoral tubercle with a yellow cap is present. The hyponotum is grey to white, and the foot sole has a black line in the mid-line; the top of the foot is white with no markings. The anus opening is placed ventrally, beautifully illustrated by Kato (2002), who also recorded the maximum size of 50 mm.

##### Distribution.

Western Pacific Ocean: **Indonesia**: Ternate (Stoffels et al. 2016 as *Phyllidia* sp.; provided georeference: −0.03108, 127.23383); Bunaken Island, North Sulawesi (Papu et al. 2022 as *Phyllidia* sp. 9; provided georeference: 1.61178, 124.76798; https://nudipixel.net/photo/00020926); Sulawesi (https://www.ryanphotographic.com/phyllidiidae.htm#picta); Wakatobi (size 49 mm: http://www.seaslugforum.net/find/4130, https://nudipixel.net/photo/00016149); **Japan** (Kato 2002: size 50 mm, 30 m depth: http://www.seaslugforum.net/find/6414; provided georeference: 33.093833, 139.768195): **Taiwan**, East China Sea (https://www.inaturalist.org/photos/430366532; provided georeference: 25.117593, 121.925725, https://www.inaturalist.org/observations/134824066; provided georeference: 25.14486, 121.80532); **Philippines**: (Gosliner et al. 2015: 282 as *Phyllidia* sp. 9, 80 m depth; Dauin https://nudipixel.net/photo/00023935); **Australia**: Western Australia (https://www.inaturalist.org/observations/110074843; provided georeference: -21.86156, 114.16379), Queensland (https://www.inaturalist.org/observations/189262927; provided georeference: −17.20932, 146.07441).

##### Remarks.

This species is so distinctive that the internet photographs listed above can be confidently assigned to *P.ovata*, first recorded more than 20 years ago. Interestingly, several images were located in files of *P.picta* Pruvot-Fol, 1957 and *P.coelestis* in both iNaturalist and NudiPixel. To further confuse matters, Yonow (2011) depicted an animal from Western Australia (Kimberley) as *Phyllidiacoelestis* (dark form) that resembles our new species in external colouration. However, the anus of this Australian specimen opens dorsally and not ventrally, and there is no black line present on the foot. Additionally, this specimen has black blotches within the white area, another feature not observed in the specimens and images of *P.ovata* sp. nov.

##### Etymology.

This species name is the diminutive form of the Latin adjective *ovatus*, -*a*, -*um* (egg-shaped) and refers to both the species’ body form as well as the coloured pattern.

### ﻿Morphology and histology

***Phyllidiafontjei* sp. nov.**

Figs 3–5

**Figure 3.**
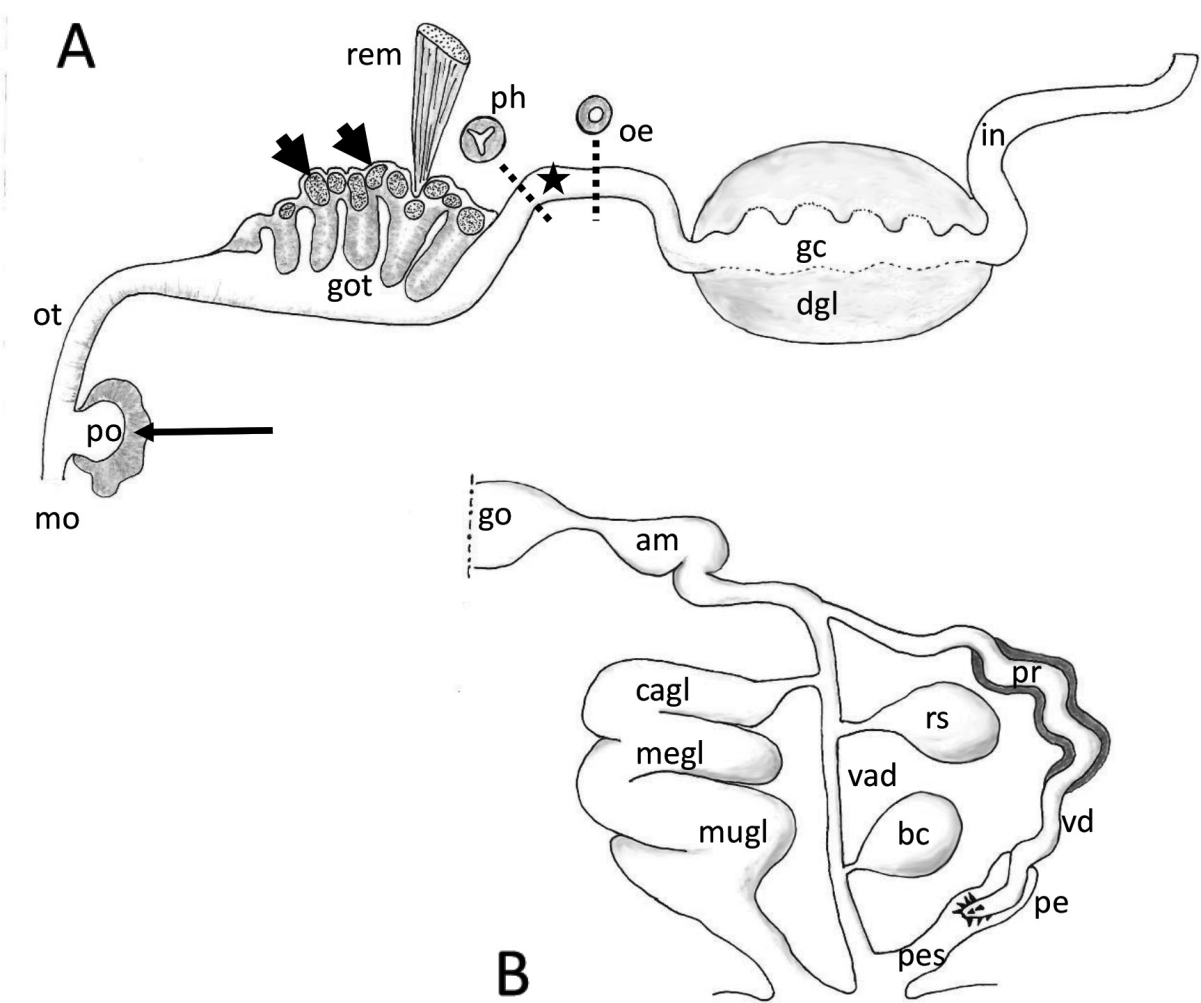
*Phyllidiafontjei* sp. nov. Schematic outlines. **A.** Longitudinal section of gastrointestinal apparatus of Phyllidia sp. nov. with cross section of pharynx and oesophagus. Arrow indicates glandular area of pouch behind mouth, arrow heads indicate the glandular lappets within the glandular folds and star indicate position of nerve ring; **B.** Genital apparatus of *Phyllidiafontjei* sp. nov. Abbreviations: am ampulla, bc bursa copulatrix, cagl capsule gland, gc gastric cavity, go gonad, got glandular oral tube, in intestine, megl membrane gland, mo mouth, mugl mucus gland, dgl digestive gland, oe oesophagus, ot oral tube, pe penis, pes penial sheath, ph pharynx, po pouch of the oral cavity, pr prostate, rem retractor muscle, rs receptaculum seminis, vad vaginal duct, vd vas deferens.

***Anatomy*.** The general plan of the gastrointestinal tract can be seen in longitudinal section in Fig. 3A, which shows a structure for *Phyllidia* that is not described or illustrated in dorsal dissection illustrations, the glandular oral pouch (Fig. 3A, po). We also redefine the misnomer “pharyngeal bulb” in the literature, and call it the glandular oral tube (GOT), consisting of glands and lappets that are extruded during feeding.

***External epithelia*.** Dorsal notum and dorsal surface of foot lined by non-ciliated pavement epithelia with submerged nuclei; gland cells could not be observed. Hyponotum lined by ciliated epithelium consisting of cuboidal cells and interspersed goblet cells with basophilic grana. Ventral foot sole lined by ciliated epithelium consisting of columnar glandular cells with submerged gland cells staining homogeneously purple in the underlying connective tissue, goblet cells with basophilic grana and many basophilic subepithelial gland cells also present and staining homogeneously purple. Gill leaflets between mantle and foot lined by ciliated epithelium consisting of cuboidal cells without gland cells.

**Figure 4.**
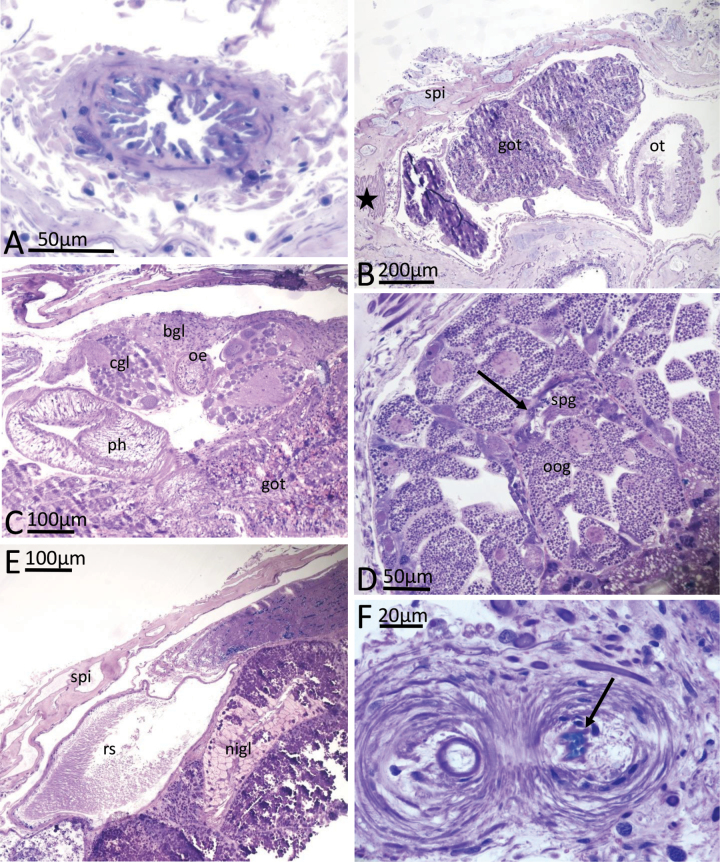
*Phyllidiafontjei* sp. nov. Images showing a selection of the histological slides. **A.** Oral tube; **B.** Oral tube and transition into oral glandular part (GOT); note dorsoventral muscle bundles (star); **C.** Pharynx emerging from GOT, and oesophagus in nerve ring; **D.** Gonad; arrow indicates area of sperm production lying in between the gonad follicles with oogonia production; **E.** Receptaculum seminis and various parts of nidamental glands with areas of exuded mucus; **F.** Cross section of penis (twice) with tiny spines (arrow), probably at the tip of the penis. Abbreviations: bgl blood gland, cgl cerebral ganglion, got glandular oral tube, nigl nidamental glands, oe oesophagus, oog oogonia in female gonad follicles, ot oral tube, ph pharynx, rs receptaculum seminis, spg spermatogonia, spi spicules in body wall.

**Figure 5.**
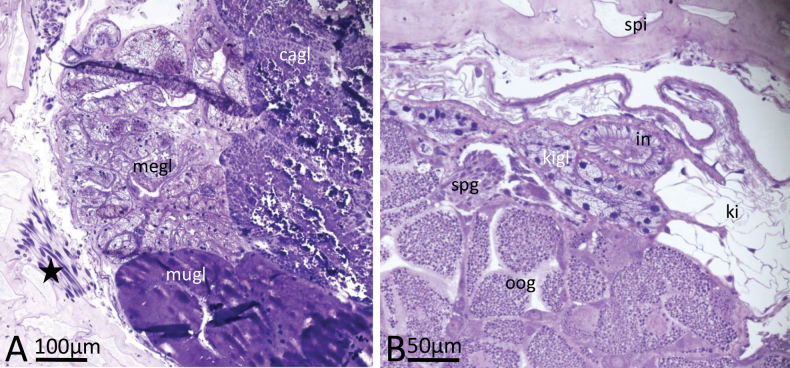
*Phyllidiafontjei* sp. nov. Images showing a selection of the histological slides. **A.** Nidamental glands; note dorsoventral muscle bundles (star) **B.** Cross section of posterior part of body with gonad containing spermatogonia and oogonia, kidney, and kidney gland. Abbreviations: cagl capsule gland, in intestine, ki kidney, kigl kidney gland, megl membrane gland, mugl mucus gland, oog oogonia, spg spermatogonia, spi spicules in body wall.

***Digestive tract*.** The mouth aperture is slit-like, in a vertical orientation, with a ciliated and folded epithelium. Subepithelial glandular follicles surround the mouth; a glandular pouch opens into the oral cavity close to the mouth. This pouch has a slightly folded epithelium and subepithelial large gland cells staining homogeneously blue. Oral tube emerging dorsally from the oral cavity above the glandular pouch and running posteriorly; epithelium of this part of oral tube highly folded, not ciliated, consisting of columnar cuboidal cells and surrounded by muscle fibres (Fig. 4A). Subsequently, oral tube opening into a large bulb-shaped and highly folded part representing the glandular part of the oral tube (GOT, Fig. 4B). GOT consisting of ciliated, tall columnar, mucus-producing cells with interspersed subepithelial gland cells, which stain homogeneously or granularly dark violet; large granular gland cells staining bluish lie in periphery of the GOT and form lappets; these extend between the glandular folds (Fig. 3A). Two large retractor muscles attach to the external lining tissue of the GOT. Pharynx emerges dorso-posteriorly from the GOT, and has a triangular lumen, surrounded by a thick layer of radial and then circular muscle fibres (Fig. 4C). Epithelium consisting of cuboidal, non-ciliated cells. Transition into oesophagus marked by position of central nerve ring. Oesophagus round in cross section with a non-glandular epithelium surrounded by longitudinal and circular muscle layers (Fig. 4C). No cuticular structures or linings were observed in the pharynx or oesophagus. Oesophagus enters large gastric cavity (stomach), which is completely embedded in the digestive gland. Stomach epithelium rarely visible on the dorsal side of the central gastric cavity, consisting of a folded layer of cuboidal, ciliated cells. Intestine arising posterio-dorsally from gastric cavity/midgut gland, forming a loop anteriorly and then leading straight towards the dorso-posterior end of animal; first part highly folded, with an epithelium of ciliated columnar cells, subsequently without internal folds (Fig. 5B). Anal pore was cut off with the tail section for molecular work and could not be observed.

***Genital system*.** Genital system outlined in Fig. 3B. Gonad lying more or less flat in the posterior part of animal, dorsally and laterally to digestive gland (Figs 4D, 5B); gonad mature with oogonia and spermatozoa in separate follicles, the latter located more in the dorsal periphery of the gonad (Fig. 5B, spg). Ampulla oval, positioned dorsally, filled with autosperm (Fig. 4E); lumen of ampulla lined by a squamous flat epithelium. Fertilisation chamber filled with sperm cells with their heads oriented towards the epithelium of fertilisation chamber. The prostatic vas deferens, as well as the ductus ejaculatorius, are convoluted. Prostate glandular epithelium of tall columnar glandular cells filled with granular contents. No subepithelial glands present. Ductus ejaculatorius ciliated but non-glandular, opening into a duct surrounded by muscle containing a slender muscular penis in a narrow penis sheath. Spines present at probably the tip of the penis (Fig. 4F, arrow); penis sheath leading into atrium surrounded by muscle that is shared with the vaginal duct. Common opening on right side between gills in anterior 1/3 of body. Vagina leading towards round, sac-like bursa copulatrix, lined by a squamous epithelium with large cells and filled with disintegrating sperm and oogonia; opening of vaginal duct into bursa copulatrix stalked with tall prismatic epithelium. Vaginal duct, lined with ciliated, cuboidal cells, running towards the oval, sac-like receptaculum seminis, surrounded by muscle fibres, located dorsally between bursa copulatrix and ampulla. Receptaculum filled with parallel-oriented sperm cells; heads of sperm cells located towards slightly folded, non-ciliated pavement epithelium of receptaculum seminis (Fig. 4E). Nidamental glands consisting of a large capsule gland, a smaller membrane gland, and a mucus gland. Capsule gland consisting of large, tall columnar cells filled with violet-staining granules. Membrane gland consisting of ciliated tubular duct with tall columnar cells filled with non-staining vacuoles. Mucus gland prominent; consisting of tall prismatic, ciliated, mucus-producing cells. Some cells of the mucus gland and capsule gland had already secreted their contents (Fig. 5A). Proximal oviduct with long cilia.

***Circulatory and excretory system*.** Muscular ventricle in thin pericardium, positioned on top of kidney. Pericardial glands in the dorsal pericardial wall were not found. Kidney flat, positioned dorsally in posterior ⅔ of the animal; nephrocytes with large non-staining vacuoles, concretions observed in several cells (Fig. 5B). Kidney tubules flanked by glandular cell complex (kidney gland) consisting of large basophilic gland cells with non-staining vacuoles. Glandular tubules ramifying between kidney tubules (Fig. 5B). This kidney gland flanks the posterior part of intestine as a mass (Fig. 5B, kigl). Renopericardial duct, syrinx, and ureter could not be described due to the tail section being removed for molecular work.

***Nervous system and sensory organs*.** Cerebropleural and pedal ganglia forming a central nerve ring surrounding the oesophagus at transition from pharynx to oesophagus (Fig. 4C). Eyes positioned dorsally, oriented laterally. Statocysts between cerebropleural and pedal ganglia containing several otoconia. Small, flat blood gland situated on top of cerebropleural complex, composed of very small cells (Fig. 4C). Rhinophores retracted, with ciliated epithelium consisting of cuboidal cells. These cells contain special vacuoles, particularly at the edges of the lamellae.

**Figure 6.**
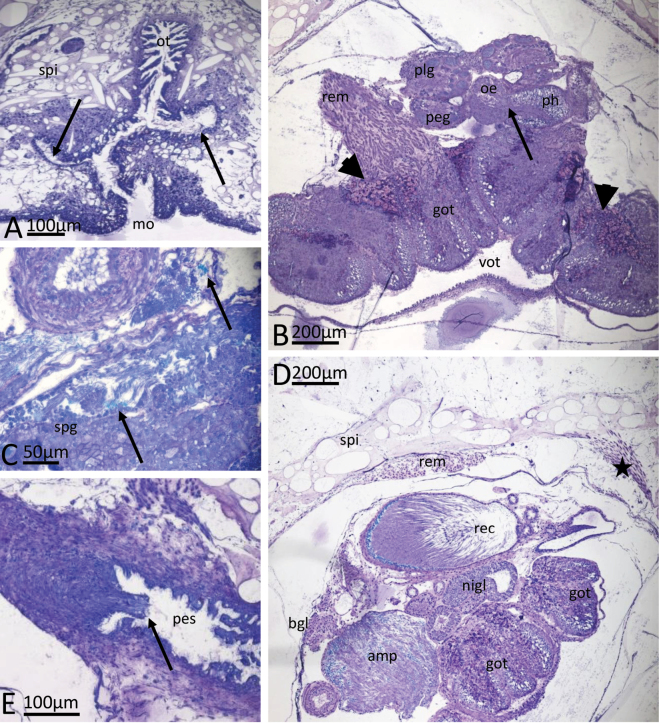
*Phyllidiaocellata* Cuvier. Images showing a selection of the histological slides. **A.** Mouth leading to non-glandular oral tube and lobed glandular pouch (arrows); **B.** Oral glands (GOT) with inserting retractor muscle and part of the nerve ring. Note the transition from pharynx into oesophagus indicated with an arrow; note the different glandular areas of the GOT, especially the violet-stained lappets (arrowheads); **C.** Gonad with spermatogonia and mature sperm (arrows); **D.** Cross section through genital system with receptaculum seminis and ampulla; insertion of retractor muscle at the peritoneal wall and dorsoventral muscle bundles in lateral notum (star); **E.** Penis with spines (arrow) in penial sheath. Abbreviations: amp ampulla, got glandular oral tube, oe oesophagus, ot oral tube, mo mouth, nigl nidamental glands, peg pedal ganglia, ph pharynx, plg pleural ganglia, pes penial sheath, rem retractor muscle, spg spermatogonia, spi spicules, vot ventral part of oral tube without glands.

***Musculature and spicules*.** One pair of bifurcated retractor muscles originating from the GOT and attached laterally to the inner sides of visceral hump. In lateral notal tissues, flanking the visceral hump, alternating prominent muscle bundles in a dorso-ventral arrangement (Figs 4B, 5A, star), similar to those in *Phyllidiaocellata* (Fig. 6D, star). Further muscle fibres throughout the notum and foot. In cross sections, spicules forming a girdle in the notal tissue around the visceral cavity (Fig. 4B). Bundles of spicules extending towards the surface along the skirt of the notum. Spicules also present in the foot.

***Phyllidiaocellata* Cuvier, 1804**

Figs 1D, 6, 7

The 16 mm living specimen was preserved in 4% formaldehyde/seawater. It is identical in colour pattern and morphology to the adults of the “classic” *Phyllidiaocellata* (Fig. 1D), as described and illustrated by Cuvier (1804). This juvenile specimen is very similar to *P.fontjei* with regard to the digestive tract, glandular parts in the epithelia, and circulatory system. However, it does differ with regards to characters in the genital system because the specimen was in the immature phase and only showed male features. In the following section, only differences from the description of *P.fontjei* are noted as well as other characters not usually observed in anatomical descriptions.

***Digestive tract*.** A glandular pouch behind the mouth was observed, similar to *P.fontjei* (Fig. 6A, arrows). The transition from the thin non-glandular part (oral tube) to the bulb-like glandular part (GOT) of the oral tube is gradual and not sudden as in *P.fontjei*, and the ventral part is without glandular folds (Fig. 6B). These conditions may be related to the ontogenetic state of the animal. Pharynx and oesophagus similar to *P.fontjei*.

***Genital system*.** The small *P.ocellata* contained a gonad exhibiting only sperm production (Fig. 6C). The nidamental glands are not developed but show a small branched system without any differentiation of the cells. Nevertheless, the ampulla is filled with autosperm and the receptaculum seminis is completely filled with allosperm, suggesting a previous copulation (Fig. 6D). The small specimen of *P.ocellata* exhibits spines in the distal part of the eversible penis (Fig. 6E).

**Figure 7.**
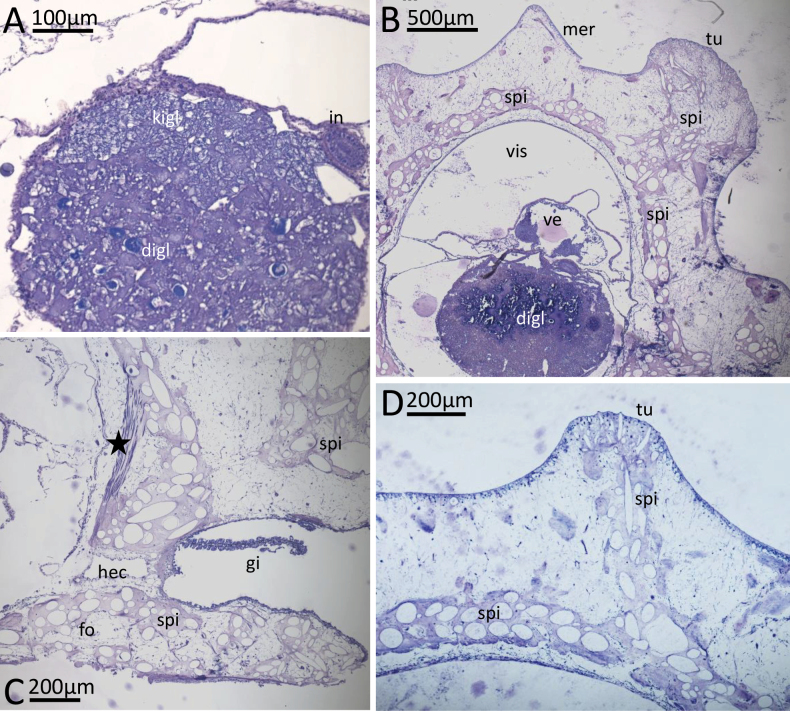
*Phyllidiaocellata* Cuvier. Images showing a selection of the histological slides. **A.** Kidney gland lying on top of digestive gland; **B.** Cross section in heart region; note the spicule girdle around the visceral cavity in the tuberculate body wall and the lack of spicules in the median dorsal ridge between the median tubercles; **C.** Cross section showing one gill lamella; note the spicules in the foot (lower spi) and the dorsoventral muscle bundles (star); **D.** Bundles of spicules branching off from the spicule girdle of the visceral cavity into a tubercle of the body wall. Abbreviations: digl digestive gland, fo foot, gi gill leaflet, hec hemolymph channel, in intestine, kigl kidney gland, mer median notum ridge in between median tubercles, spi spicules, tu tubercle, ve ventricle, vis visceral cavity.

***Circulatory and excretory system*.** Kidney forming a sac-like structure on top of digestive gland. Kidney gland with faint bluish-staining glandular cells, forming a thick layer covering the posterior part of the digestive gland (Fig. 7A).

***Musculature and spicules*.** In cross section, spicules form a girdle in the notal tissue around the visceral cavity (Fig. 7B, C). Bundles of spicules extend towards the surface of the notum and reach into the distinct tubercles (Fig. 7B, D).

## ﻿Discussion

**Figure 8.**
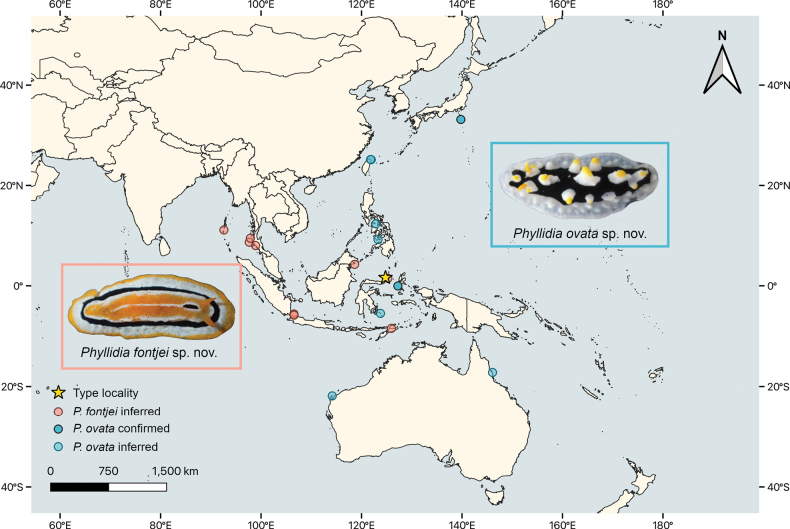
Distribution map of the two new species described in this paper. Type locality (star) is the same for both species (Bunaken Island, North Sulawesi). Confirmed records are inferred by sequence analyses and one photographic record of the ventral side, whereas unconfirmed findings are inferred only from images available on the internet.

In contrast to other species of *Phyllidia*, such as *P.ocellata*, *P.coelestis*, or *P.elegans* Bergh, 1869, the two new species described herein have distinctive colouration and patterning that allow an identification in both the field and of photographs. However, the ventral side should always be examined (in all phyllidiids), to establish the presence of a black line and the ventral anus in the case of *P.ovata* sp. nov. By re-evaluating available photographs from websites, we are able to extend the range of distribution for both species beyond their holotype localities (Fig. 8). Taking the available information into consideration, we can conclude that both species are rather rare compared to other Indo-Pacific *Phyllidia* species. While *P.ovata* sp. nov. is restricted to a narrow band in the Western Pacific from Japan south to Taiwan, Indonesia, and the Philippines with the southernmost records on both sides of Australia., the distribution of *P.fontjei* sp. nov. extends west from Indonesia to the edge of the eastern Indian Ocean, with images recorded from western Thailand and the Andaman and Nicobar Islands.

We only found one study covering histology in *Phyllidia* (Permadani and Retnoaji 2018) that presents some sections on the epithelia of *Phyllidiacoelestis*. The most extensive study on *Phyllidiaflava* from the Mediterranean Sea was by Wägele (1985) who noted that the posterior glandular part of the oral tube has nothing to do with the pharynx and did not use the misleading term “pharyngeal bulb”. We here introduce the term “glandular oral tube” or GOT.

Somewhat surprisingly, despite differences in distribution and sizes, the histological observations on *P.fontjei* and *P.ocellata* differ only in few aspects from *P.flava* and thus confirm the many morphological studies on *Phyllidia* species, which show only small interspecific anatomical differences such as in the reproductive organs (Domínguez et al. 2007) and in the presence or absence of various types of black pigment on the oral tube and anal papilla, and shapes of the anal papilla (Yonow 1996, 2011; Domínguez et al. 2007; pers. obs. HW, NY).

*Phyllidiaflava* is the largest of the three species that have been sectioned: *P.flava* was ≤ 30 mm alive, and *P.ocellata* and *P.fontjei* were both only 16 mm. The observation of a glandular pouch present behind the mouth in the three species is interesting and has not been observed or mentioned in dissections studies. The function of this glandular pouch is not known. The transition from the thin non-glandular part of the oral tube to the GOT is rather sudden in *P.fontjei*, whereas the transition is more gradual in *P.ocellata* and *P.flava*. For all three specimens, and indeed other species of *Phyllidia*, the glandular lobes are concentrated inside the oral bulb and everted for external digestion, as shown by Yasman (2003) and van Alphen et al. (2010: fig. 1b, c). We hypothesise that the lateral muscle bundles arranged serially on both sides in the body wall (Fig. 7C) and also observed by Wägele (1985) in *Phyllidiaflava* are responsible for the extrusion of the GOT. When they contract, the visceral hump is squeezed with an increase of haemolymph pressure towards the anterior. The two retractor muscles attached to the inner surface of the dorsal hump retract the everted glands and lappets after feeding. More information on the number and structure of the glands and lappets in the GOT are needed to reveal this character as a putative species-specific feature; drawings of the structures were given for *Phyllidiarueppelii* (Bergh, 1869) by Yonow (1996: fig. 12) and illustrated for *P.varicosa* Lamarck, 1801 by van Alphen et al. (2010: fig. 1b, c) with photographs also of the pattern remaining in the surface of the sponge Axinyssaaff.variabilis.

The kidney gland was first described in *P.flava* and also occurs in *P.fontjei* sp. nov. and *P.ocellata* and may in fact be present in all species of *Phyllidia*. Wägele (1985) described the gland as tubes extending between the kidney tubules and lobes in *P.flava*. In *P.fontjei* and *P.ocellata*, it does not extend between parts of the kidney but forms a layer (*P.ocellata*) or a larger mass (*P.fontjei*) on top of the posterior digestive gland. The function of the kidney gland remains unknown and cannot be observed in dissections.

The presence of pericardial glands in the dorsal part of the pericardial wall is described in detail for *P.flava* by Wägele (1985), but these glands were not observed in either *P.fontjei* sp. nov. or *P.ocellata*. The absence might be due to smaller body size but needs further investigations.

The analysis of the reproductive system, especially the condition of the gonad and the nidamental glands, showed that *P.flava* and *P.fontjei* were both functioning reproductive specimens while *P.ocellata* was clearly a juvenile. In the first two specimens, the gonad exhibited sperm and oogonia formation, whereas in *P.ocellata* the gonad only contained spermatocytes, indicating a functional male phase. In the latter, the nidamental tubes, responsible for building up the complicated structure of the egg mass around the fertilized eggs, did not show any glandular areas, where as in the similarly sized *P.fontjei* specimen the nidamental gland was subdivided into the typical capsule, membrane, and mucus glandular areas of other nudibranchs (Klussmann-Kolb 2001). The areas in the nidamental glands of cells containing no mucus reveal evidence of the formation and release of egg masses, another indication of its full maturity in the female phase. *Phyllidiaocellata* had a filled receptaculum seminis that indicated a previous copulation.

*Phyllidiaflava* and *P.ocellata* are both characterised by dorsal tubercles, in which bundles of spicules are present. *Phyllidiafontjei* is rather smooth without distinct tubercles; however, spicule bundles from the internal side of the notum extend towards the external surface of the notum. The presence of these spicule bundles indicates a reduction of external tubercular structures that were probably present in the putative predecessor of this species. This hypothesis is also supported by molecular phylogenetic analyses, which show a close relationship of *P.fontjei* to tuberculate species like *P.ocellata* (Papu et al. 2022). Chang et al. (2013) have shown that the arrangement of the spicules could not be used to distinguish between the species.

Although there are some differences revealed by histological investigations in the three species, the most important conclusion that can be drawn from this study is their similarities and that, finally, the gross anatomy of *Phyllidia* does not vary significantly between species except that details of pigmentation of the peritoneum, oral tube, and pharynx can vary. From this work, we can also surmise that *P.fontjei* is a small species due to its maturity, compared to *P.ocellata* that can grow up to a size of 50 mm (41.2 mm preserved, Domínguez et al. 2007), or *P.flava* that reaches 45 mm (Cattaneo-Vietti et al. 1990) and *P.ovata* up to 50 mm (Kato 2002).

## Supplementary Material

XML Treatment for Phyllidia fontjei

XML Treatment for Phyllidia ovata

## ﻿Additional information

### Author ORCIDs

Heike Wägele https://orcid.org/0000-0001-6899-0336

Lina Raubold https://orcid.org/0000-0002-3041-6416

Adelfia Papu https://orcid.org/0000-0001-7790-9405

Nani Undap https://orcid.org/0000-0001-5846-1211

### Data availability

All of the data that support the findings of this study are available in the main text.
